# Super-radiant mode in InAs—monolayer–based Bragg structures

**DOI:** 10.1038/srep14911

**Published:** 2015-10-12

**Authors:** G. Pozina, M. A. Kaliteevski, E. V. Nikitina, D. V. Denisov, N. K. Polyakov, E. V. Pirogov, L. I. Goray, A. R. Gubaydullin, K. A. Ivanov, N. A. Kaliteevskaya, A. Yu. Egorov, S. J. Clark

**Affiliations:** 1 Department of Physics, Chemistry and Biology (IFM), Linkoping University, S-58183 Linkoping, Sweden; 2 St-Petersburg Academic University Khlopina 8/3, 194021, St-Petersburg, Russia; 3 Ioffe Physical-Technical Institute of Russian Academy of Science, Polytechnicheskaya 26, 194021, St-Petersburg, Russia; 4 ITMO University, Kronverkskiy pr. 49, 197101 St. Petersburg, Russia; 5 Department of Physics, Durham University, South Road, Durham, UK DH1 3LE

## Abstract

We report direct experimental evidence of the collective super-radiant mode in Bragg structure containing 60 InAs monolayer-based quantum wells (QWs) periodically arranged in GaAs matrix. Time-resolved photoluminescence measurements reveal an appearance of the additional super-radiant mode, originated from coherent collective interaction of QWs. This mode demonstrates a super-linear dependence of the intensity and radiative decay rate on the excitation power. The super-radiant mode is not manifested in the case if only a small number of QWs is excited.

Coherent interaction of an ensemble of emitters and the electromagnetic field can modify optical properties of emitters. This effect was theoretically investigated already in the 1950 s by Dicke^1^, who considered a system of emitters in the case when a spatial separation between them is much smaller than the wavelength of light. It was shown, that a collective super-radiant (SR) mode can be formed and that the rate of spontaneous emission is proportional to the square of the number of emitters. Such interaction can occur even if the emitters are separated by macroscopic distances but are arranged periodically with a period corresponding to the half of the emission wavelength, i.e. forming Bragg structure, as it was shown in the later theoretical work^2^. The coherent interaction of the emitters has been demonstrated experimentally in microcavities^3^, atomic systems^4^, quantum dots^5^, and for Bragg multiple quantum wells (BMQW)^6^,^7^. In the latter case, the observed excitonic Bragg reflection could reach 80%. A large variety of interesting effects associated with formation of Bragg polaritons can be also monitored when BMQW is incorporated into dielectric Bragg reflectors^8^,^9^.

Besides reflection studies, direct experimental results concerning light-induced collective effects in such BMQW have been demonstrated by degenerate four-wave mixing measurements on excitons in GaAs/AlGaAs MQW containing several identical QWs separated by equivalent distances^10^ or using Brillouin scattering by acoustic phonons^11^. The latter experiment was performed on the structure containing 40-periods of GaAs/AlAs layers with respective thicknesses of 17.1 and 7.5 nm. A weak signature of the radiative inter-well coupling has been observed in photoluminescence (PL), though only for the InGaAs/GaAs MQWs with the barrier thicknesses nearly satisfying Bragg resonance^12^. Taking into account that BMQW structures demonstrate ultrafast optical switching^13^ such systems are very promising for different future optoelectronic devices.

In this work, we present the first experimental results of light emission via collective SR mode of BMQW structure, formed by InAs single monolayers confined in GaAs matrix. Nonlinear properties of the system are favorable for different potential applications such as optical logic devices and optical switches. We report a super-linear dependence of the SR mode on the pumping intensity, which means a higher probability of the spontaneous emission. The observed effect is a prerequisite for creating a cavity-less laser on SR mode with low threshold and high efficiency.

## Results and Discussion

There are several technological advantages for the structures based on InAs single monolayers compared to MQW fabricated using II-V solid solutions. First, there is no contrast of background dielectric constant between InAs monolayer and GaAs matrix and, thus, there is no associated with that contribution to the photonic band structure. Second, in such binary compound heterostructures there are no local fluctuations in chemical composition. Additionally, the elastic energy of the strained monolayer is rather moderate and will not induce dislocations and/or cracking of the structure. At the same time, the confinement of electrons and holes in InAs monolayers is essential.

**Figure 1:**
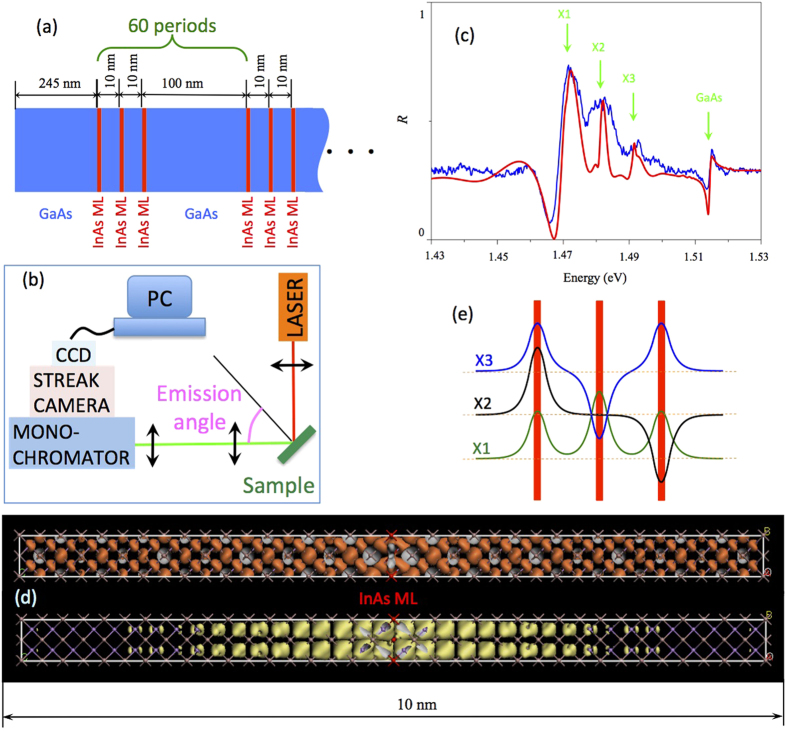
(**a**) Schematic drawing of the structure. (**b**) Sketch of the experimental setup. The angle of emission was varied by the rotation of the sample. (**c**) Reflection spectrum measured at 5 K taken at normal. Experimental data are shown by the blue line; results of the modeling according to Eq. (1) are shown by the red line. Arrows indicate the excitons resonance energies, used for fitting. (**d**) Probability density for electron (upper) and hole (lower) states localized at the InAs monolayer calculated by density functional theory. InAs monolayer confined by the GaAs crystalline matrix in placed at the center of the structure. (**e**) Schematic illustration of the coupled exciton localized in the three monolayer InAs quantum wells. The coupling leads to the triple exciton resonance feature in the reflection.

For the studies we have used a 60 periods structure illustrated in Fig. 1(a), where the active regions were formed by three InAs single monolayers. The details of the structure design and growth recipe together with experimental set-up (see Fig. 1(b)) are provided in the method section. This high quality BMQW structure demonstrates several intensive peaks of excitonic reflection, shown in Fig. 1(c). It can be seen that there is a feature corresponding to GaAs exciton at 1.515 eV, and the triple-peak structure associated to excitons, localized on InAs monolayers. Fitting of the experimental reflection spectrum allows to obtain parameters of the X1, X2, and X3 excitons, respectively, which are summarized in Table 1.

Electron and holes experience a localization on the InAs monolayer embedded in the GaAs matrix and the localization energy is 16 meV for electron and 19 meV for the hole, respectively. Thus, the energy of the exciton localized at isolated InAs ML is 1.480 eV. Fig. 1(d) shows the wavefunction of the localized electron and hole states calculated using ab-initio density-functional theory approach. It can be seen that the hole state is localized and the localization length of the hole wavefunction is ~1 nm. Due to a small electron effective mass in GaAs electron localization length is much larger, about 15 nm. Therefore, since the distance between nearest InAs MLs is 10 nm, electron can be considered as a free particle. On other hand, holes are localized but the wavefunctions of the holes localized in neighboring InAs MLs overlap, which leads to the appearance of the triplet structure in the eigen-mode spectra. This triplet schematically shown in Fig. 1(e), is responsible for peculiar shape of the reflection spectra shown in Fig. 1(c).

**Figure 2:**
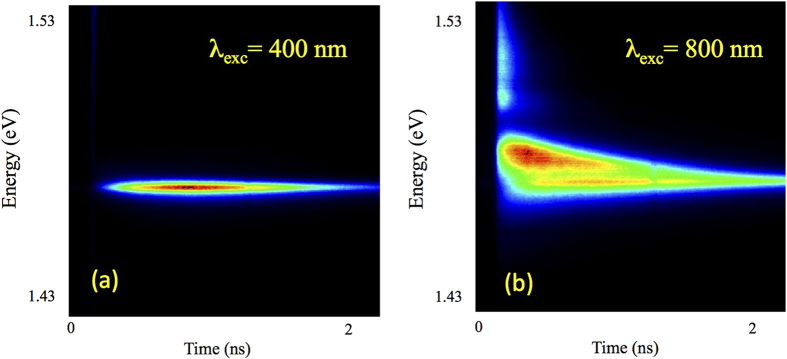
Low-temperature (5 K) TRPL images taken under excitation by the laser with the wavelength of 400 nm (a) and 800 nm (b). The emission angle is 40° in both cases. An average excitation power was kept to ~30 mW in both cases.

Fig. 2(a) shows low-temperature time-resolved PL (TRPL) image taken at the emission angle of 40° under excitation by a laser with the wavelength of 400 nm. Absorption coefficient of GaAs for this wavelength is 10^5^ cm^−1^, therefore only few InAs monolayers adjacent to the sample surface are excited. It can be seen, that there is only one emission line, corresponding to the exciton state X1. However, the TRPL behavior changes dramatically when the structure is excited by the laser with the wavelength of 800 nm (see Fig. 2(b)), when the light can penetrate to the distance up to 10 μm into GaAs. In this case, the whole entire structure and all InAs monolayers are excited. It leads to the appearance at higher energies of the additional intensive emission line, caused by collective coherent interaction of the individual excitons with electromagnetic field, i.e. of the SR mode. It is interesting to note that the SR mode decays much faster than the X1 mode. Also, it is clear, that the spectral width of the X1 mode shown in Fig. 2(a), when only few monolayer quantum wells are excited, and is comparable to the width of the X1 mode shown in Fig. 2(b), when all 180 monolayers are excited. The latter confirms an extremely high crystalline quality and the uniformity of the quantum wells based on binary InAs monolayers forming the BMQW structure.

**Figure 3:**
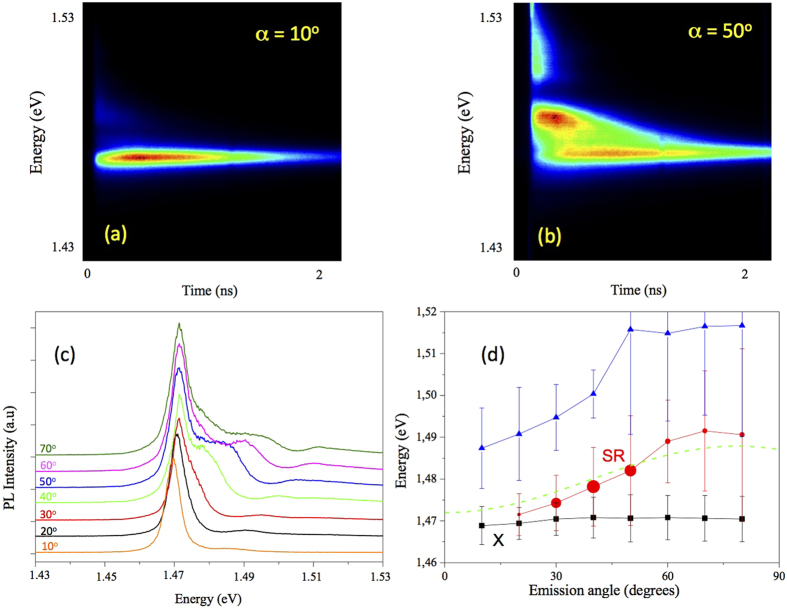
TRPL image for the emission angle of 10 degrees (**a**) and 50 degrees (**b**). (**c**) Time-integrated PL spectra for different emission angles indicated for each spectrum. (**d**) Angular dependence of the photon energy of the X1 and SR lines. Vertical bars indicate the full width at half maximum (FWHM) of the lines obtained by a fitting of the spectra shown in (**c**). The dashed line shows the angular dependence of the SR mode energy obtained using Eq. (6).

The angular dependence of the PL properties is illustrated in Fig. 3. It can be seen in Fig. 3(a) that for small emission angles, the SR mode is merged with the X1 mode. For the emission angle of 50 degrees, the SR mode becomes most pronounced. Note here, that the photon energy of the SR mode varies in time. Such variation is likely caused by a temporal change of non-equilibrium charge carriers, which leads to a temporal change of the effective refractive index of the structure. Further, the effective refractive index could be additionally varied in time due to Kramers-Kronig relations, i.e. when an excitation pulse leads to a substantial variation of the absorption coefficient spectrum. Thus, integration over the time results in a substantial broadening of the SR emission line. An increase of the angle does not lead to a noticeable variation of the spectral position of the exciton mode X1, while the photon energy of the SR mode increases with increasing angle, and satisfy the Bragg condition as shown by the dashed line in Fig. 3(d).

**Figure 4:**
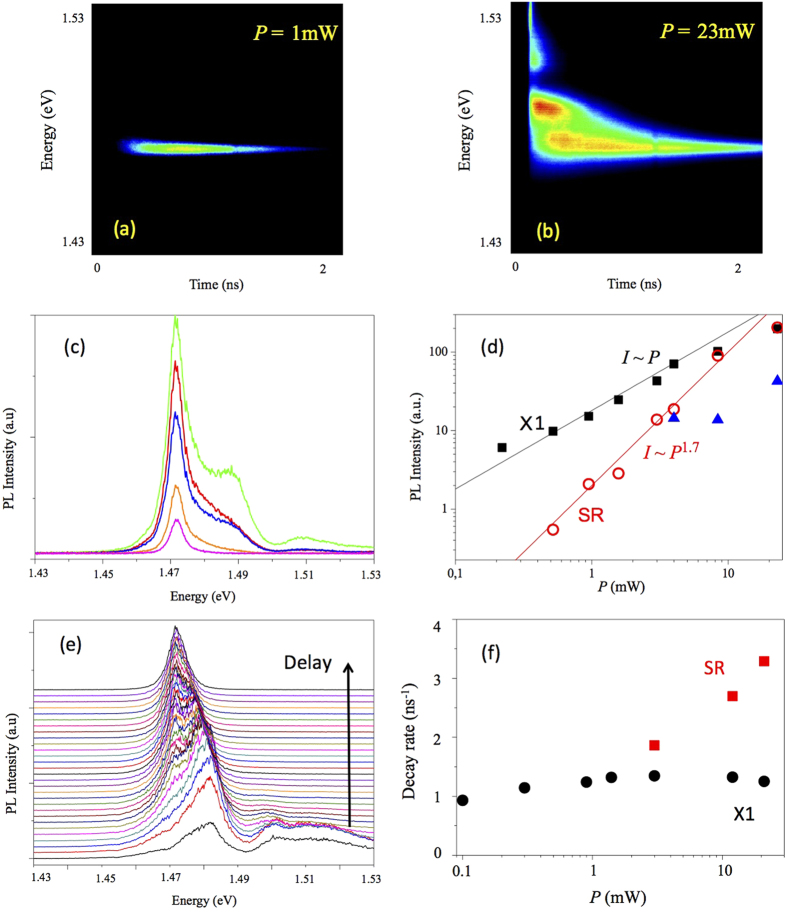
Time resolved PL images taken at the emission angle of 50° for the excitation power of 1 mW (a) and 23 mV (b). (**c**) Time-integrated PL spectra shown for different excitation powers 1 mW, 3 mW, 8 mW, 16 mW and 23 meV. (**d**) Dependence of the integrated PL intensity for the SR and X1 modes on pumping power *P*. (**e**) Time-resolved PL spectra shown for different delays, delay time between each spectrum is 50 ps. (**f**) Dependence of the PL decay rate on the pumping power for normal emission for the SR an X1 modes.

Moreover, the X1 and SR modes have a different response to the variation of the excitation power, as illustrated in Fig. 4. For the small excitation power, there is no emission associated with the SR mode. Increase of the excitation power induces a linear growth of the emission intensity of the X1 mode, while the SR mode demonstrates a super-linear growth. Similar super-linear behavior was predicted for collective SR mode by Dicke^1^. PL dynamics for the SR and X1 modes is also different, as shown in Fig. 4(e, f), respectively. For the SR mode, the PL delay between the excitation pulse and the peak intensity of the luminescence is much shorter than for the X1 mode, which is an evidence of the stimulated scattering^14^. Also, the radiative decay rate for the SR mode demonstrates a significant increase with increasing excitation power.

In summary, we have experimentally demonstrated existence of the collective SR mode originated from the coherent interaction of BMQW structure, in which the InAs MLs inside the GaAs matrix serve as quantum wells. The SR mode manifests itself only when a large number of quantum wells is excited. The SR mode demonstrate super-linear dependence of the PL intensity and decrease of radiative lifetime with increasing excitation power. The PL properties demonstrated by the BMQW structure make such systems promising for the realisation of novel types of lasers, switches, and other optoelectronic devices.

## Methods

### Experimental

The sample was grown by molecular beam epitaxy using Riber 49 growth chamber on (100) GaAs substrate. The growth rate has been controlled *in-situ* using high energy electron diffraction. The active areas have been composed by three InAs monolayers confined in GaAs matrix. The spatial separation between individual monolayers was 10 nm. There were 60 periods of such active areas in the structure, separated by undoped GaAs layers with the thickness of 100 nm. The Bragg structure was designed to satisfy Bragg condition for the wavelength corresponding to the emission line in the individual InAs monolayer quantum well confined inside the GaAs barriers at 5 K. Thus, the peak photon energy for the emission line is 1.48 eV, while the optical thickness of the period in the Bragg structure is 418 nm.

The thickness of the GaAs cap layer was chosen to provide a matching of the antinodes of the electric field of the SR mode to the active areas. The part of the GaAs cap layer near the sample surface was doped in order to avoid the GaAs excitonic reflection feature from the surface of the sample. The growth was done with rotating substrate to provide uniformity of the structure.

PL and TRPL was excited with 800 nm wavelength from a Ti: sapphire femtosecond pulsed laser with a frequency of 75 MHz. The average power density of the laser was 100 W/cm^2^ as a maximum. A Hamamatsu syncroscan streak camera with a temporal resolution of ~20 ps was used for detection of the TRPL signal. The samples were placed in a liquid-He cryostat providing temperatures in the range 2–300 K. The angle of emission was tuned by rotating the sample. We point out here that even though the angle of incidence of the pumping laser is also varying it does not significantly affect the results of experiment. When the incidence angle is varied from 0 to 70 degrees, the propagation angle change within the structure is less than 15 degrees. The pumping is non-resonant; thus, the change of the propagation angle of the pumping radiation leads to only a small decrease of its penetration within the structure.

### Modelling

The reflection spectra were modeled using transfer matrix method^15^. The reflection coefficient *r* at normal incidence is defined by transfer matrix through the whole structure, 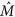:


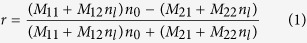


where *n*_0_ and *n*_*l*_ are the refractive indices of the first and the last semi-infinite media surrounding the structure, respectively. The matrix 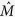 is the product of the transfer matrices of each layer. The transfer matrix for a monolayer reads:


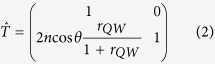


where the excitonic amplitude reflection coefficient of the monolayer *r*_*QW*_ is given by:


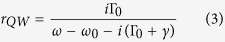


Where, Γ_0_ and *γ* are the radiative and non-radiative damping of an exciton respectively.

_The transfer matrix through the layer of the thickness **d** with the refractive index **n** can be written:_


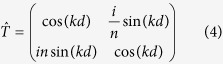


The energies of the exciton and holes levels and corresponding wavefunctions were obtained by *ab-initio* calculation using density functional theory DFT approach^16^,^17^,^18^.

The Bragg condition is written as:


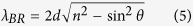


where λ_*BR*_ is the wavelength in the vacuum and *n* is the effective refractive index of the layered media and θ is the angle of propagation of the wave in a layered media^19^, which leads to an angular dependence of the phonon energy of the SR:


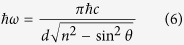


## Additional Information

**How to cite this article**: Pozina, G. *et al.* Super-radiant mode in InAs—monolayer–based Bragg structures. *Sci. Rep.* **5**, 14911; doi: 10.1038/srep14911 (2015).

## Figures and Tables

**Table 1:** Parameters of exciton, obtained from fitting of the reflection spectrum.

	X1	X2	X3
*ћω*_0_(eV)	1.4712	1.482	1.491
Γ_0_(meV)	0.04	0.02	0.015
γ (meV)	0.7	0.7	0.7

Here, *ћ*ω_0_ corresponds to the exciton resonance energy; Γ_0_ and γ are radiative and non-radiative damping of exciton, respectively.
